# Factors Associated with AI Use in a Norwegian Sample

**DOI:** 10.3390/bs16040537

**Published:** 2026-04-03

**Authors:** Sebastian Oltedal Thorp, Lars M. Rimol, Martine Klock Fleten, Simen Kristoffer Berg Hoel

**Affiliations:** Department of Psychology, Norwegian University of Science and Technology (NTNU), 7034 Trondheim, Norway; lars.morten@ntnu.no (L.M.R.);

**Keywords:** artificial intelligence, AI use, workplace, education, strengths-based leadership, gender, logistic regression, Norway

## Abstract

This study examined factors associated with self-reported workplace artificial intelligence (AI) use in a Norwegian employee sample (N = 196). Hierarchical logistic regression tested whether education, job sector, gender, age, leadership role, strengths-based leadership (SBL), perceived work training, and work engagement were associated with AI use. Higher education, employment in knowledge-intensive sectors, male gender, and higher perceived SBL were associated with greater odds of reporting AI use, whereas age, leadership role, general work training, and work engagement were not. The study’s contribution is exploratory: it suggests that organizational context, and particularly perceived SBL, may add explanatory value beyond demographic and sectoral differences in a Norwegian setting. Because the study relies on a cross-sectional convenience sample, a binary self-report measure of AI use, and models affected by sparse cells, the findings should be interpreted as tentative associations rather than causal evidence about AI adoption.

## 1. Introduction

Following the rapid emergence of increasingly capable generative AI (GenAI), understanding the drivers of workplace AI use has become a critical research priority. Recent studies also show that employee responses to AI-related change are shaped by intentions and behavioral adaptation in digital-intelligence transformation and human–robot collaboration settings (Wu et al., 2025a, 2025b). Reported AI use nevertheless remains uneven across sectors, demographic groups, and organizational contexts. At the same time, effective use of AI may yield substantial gains in efficiency and workforce development, with evidence converging on task-level performance improvements of roughly 5–25% across knowledge-work activities (Choi et al., 2023; Cui et al., 2026; Dell’Acqua et al., 2023; Merali, 2024; Peng et al., 2023).

In this manuscript, we distinguish between AI adoption as a broader process of acceptance and implementation, and AI use as employees’ self-reported current use of AI at work. Because the dependent variable in the present study captures whether respondents report using AI in job-related tasks, our empirical focus is workplace AI use. Prior literature nevertheless suggests that education, sector, age, and gender are relevant correlates of such use. Higher levels of education are associated with greater uptake, reflecting both digital literacy and the alignment of AI tools with knowledge-intensive tasks (Brey & van der Marel, 2024). Sectoral patterns similarly show that use is concentrated in information-intensive industries, while it remains more limited in skill-based sectors (McElheran et al., 2024). Younger workers also report higher use than older cohorts in Norway and Denmark (Jacobsen & Erenbjerg, 2025; Rybalka, 2024). Gender differences are also reported across surveys, with men often reporting higher confidence and higher use than women (Aldasoro et al., 2024; Dorta-González et al., 2024).

Organizational factors may further condition workplace AI use. Research on innovation consistently shows that leadership facilitates the uptake of new technologies (Damanpour, 1991; Damanpour & Schneider, 2006; Hameed et al., 2012). Recent evidence extends this pattern to AI, suggesting that supportive leadership behaviors increase trust in and willingness to use AI tools (Hu et al., 2025; Zárate-Torres et al., 2025). One potentially relevant approach is strengths-based leadership (SBL), which emphasizes recognizing and developing employees’ strengths. SBL may matter because it has been linked to self-efficacy, confidence, and constructive experimentation (Ding & Quan, 2021; Van Woerkom et al., 2016; J. Wang et al., 2023). In the present study, we treat self-efficacy and related psychological pathways as plausible but untested mechanisms rather than as demonstrated mediators.

Beyond leadership, broader organizational resources may also condition AI use. Access to job-embedded training is often discussed as a facilitating condition for technology integration, because it may strengthen employees’ confidence and capacity to apply new tools in their daily work (Boothby et al., 2010; Hu et al., 2025; Molino et al., 2020). Work engagement may also be relevant, although the direction of the relationship is less clear: engaged employees may be more willing to experiment with innovations, but AI use may also affect engagement over time (Liu & Li, 2025).

Yet, the incremental explanatory value of leadership, training, and engagement beyond sociodemographic and sectoral factors remains insufficiently understood. Our contribution is therefore narrower than a general theory of AI adoption: using a Norwegian employee sample, we examine whether these organizational factors are associated with self-reported workplace AI use over and above education, sector, gender, age, and leadership role. We treat job sector as a coarse proxy for task environment rather than a direct measure of occupation or task content, and we interpret all findings as associations rather than causal effects. Consistent with prior work, we expected positive associations for higher education (H1) and knowledge-intensive sectors (H2), a male advantage in reported AI use (H3), a negative association with age (H4), and positive associations for SBL (H5), leadership role (H6), perceived general work training (H7), and work engagement (H8).

## 2. Related Works and Study Objectives

### 2.1. Education

Research consistently shows that education is positively associated with the adoption of AI tools in the workplace. OECD survey data indicate that workers with tertiary education are substantially more likely to use AI than those with lower educational attainment, largely because AI adoption is concentrated in occupations requiring complex, non-routine cognitive tasks (Lane, 2024; Lane et al., 2023). Bick et al. (2024) find that in the U.S., the share of employees using GenAI at work was 39.4% among bachelor’s degree holders and 40.9% among those with master’s degrees, compared to 19.6% among those without a college degree. Complementary findings by Kacperski et al. (2025), based on German web-tracking data from November 30, 2022, through the first 11 months after launch, show that higher education significantly predicts ChatGPT use (OR = 1.23).

### 2.2. Job Sector

AI use differs across sectors, but the underlying mechanisms likely operate at multiple levels: industry context, occupational composition, and task content. The focal predictor in the present study is sector, which provides only a coarse contextual proxy. Sectors rich in technical talent and digital infrastructure, such as IT services, media, and telecommunications, are consistently early adopters of AI (Lane, 2024). Workforce composition accounts for much of the variation in use across sectors, as more educated and digitally proficient workers tend to be earlier adopters (Brey & van der Marel, 2024). Eurostat data similarly show that by 2024, AI adoption exceeded 25% in information, communication, and professional services, but remained in single digits in construction and hospitality (Eurostat, 2024).

At the occupational level, use is highest in knowledge-intensive roles. By late 2024, 28% of U.S. workers reported using generative AI, with nearly half of computer and mathematical scientists (49.6%) and managers (49.0%) reporting use, compared to around 40% in business, finance, and education, and below 25% in routine office support, blue-collar, and personal-service roles (Bick et al., 2024). Higher education mirrors this divide, with STEM students and faculty reporting higher adoption and comfort with GenAI than non-STEM peers (Freeman, 2025; J. Kim et al., 2025; Parviz, 2024).

Task-level evidence helps explain why these differences emerge. Workers primarily use AI for cognitive activities such as writing, information search, and summarization, whereas physical tasks remain less supported (Bick et al., 2024). Usage data from large AI systems similarly show concentration in software, writing, communication, and information-processing tasks (Handa et al., 2025; Tomlinson et al., 2025).

### 2.3. Gender

A synthesis of 18 studies concluded that there is a near-universal gender gap across regions and sectors, with men reporting higher rates of generative AI use than women (Otis et al., 2024). Norwegian statistics similarly report higher use among men than women (SSB, 2025). This pattern is corroborated by national statistics in Denmark, the United Kingdom, and broader international surveys (Department for Science, Innovation & Technology, 2024; Jacobsen & Erenbjerg, 2025; Lane et al., 2023). These disparities are not necessarily reducible to occupational exposure alone and may also reflect differences in confidence, anxiety, norms, and access to relevant training opportunities (Aldasoro et al., 2024; Chien & Kim, 2024; Conde-Ruiz et al., 2024; Rana et al., 2024; Russo et al., 2025). Because the survey item captured self-reported gender rather than biological sex, we refer to gender throughout the manuscript.

### 2.4. Age

Research demonstrates a clear age gradient in AI use, a pattern supported by academic studies and large-scale surveys. In Korean enterprises, age was a significant negative predictor of generative AI use (Y. Kim et al., 2024). More broadly, some studies suggest that older workers report lower AI-related uptake or intention (Dingel et al., 2024; Fazi et al., 2025). Survey data across 11 European countries show that 36% of workers aged 18–34 report using AI at work versus 18% of those aged 55–74 (Da Silva & Weißler, 2025). Norwegian statistics show a similar pattern, with substantially higher AI use among younger than older respondents (SSB, 2025).

Several mechanisms may underlie this pattern. Older users often report higher perceived effort and greater technology anxiety, whereas younger users more often emphasize usefulness, ease of use, and trust (Grassini et al., 2024; Møgelvang & Grassini, 2025; A. Wang et al., 2024; Yu & Chen, 2024). At the same time, the literature suggests that education may partially mitigate age-related barriers (Draxler et al., 2023; Kacperski et al., 2025).

Because the current study does not model age-by-education interactions, we treat this literature as contextual rather than as a tested mechanism. Our age hypothesis is therefore modest: we expect lower self-reported AI use in older age groups, while recognizing that this association may be attenuated once education, sector, and organizational factors are taken into account.

### 2.5. Leadership

In exploring leadership, this study focuses on Strengths-Based Leadership (SBL). Emerging from positive psychology, SBL centers on identifying, developing, and applying employees’ strengths at work (Miglianico et al., 2020; Peterson & Seligman, 2004). Rather than focusing on deficits, SBL emphasizes individual capabilities and the alignment of work with those capabilities (Straume & Vittersø, 2012). Evidence suggests that SBL can strengthen self-efficacy and work engagement (Ding & Quan, 2021; J. Wang et al., 2023), while the broader leadership literature shows that supportive and transformational leadership styles often facilitate technology implementation and digital change (Anderson & Dexter, 2005; Flanagan & Jacobsen, 2003; Khirfan et al., 2024; Jeong & Jeong, 2025; Patnaik & Bakkar, 2024). We use this broader literature to motivate, but not equate, SBL with other leadership approaches. In the present model, SBL is expected to show an incremental association with AI use, although its overlap with perceived training support and engagement makes this an empirical question rather than a foregone conclusion.

### 2.6. Training

Training is often framed as an important enabling condition for AI use because it may build AI literacy, reduce effort expectancy, and increase confidence in using new systems (Ke et al., 2025; Venkatesh et al., 2003). Prior studies also suggest that training can support knowledge sharing and may reduce some demographic disparities in use when employees receive equal access and support (Chien & Kim, 2024; Hu et al., 2025; Qazi et al., 2025). However, the measure available in the present study captures perceived adequacy of general job training rather than AI-specific training or AI competence. Our hypothesis is therefore intentionally cautious: perceived general training support may be positively associated with AI use, but any null finding cannot be taken as evidence that AI-specific training is unimportant. Some of the cited training literature concerns performance improvement or capability development rather than direct evidence of routine workplace AI use; we therefore use it only to motivate training as a potentially relevant enabling condition.

### 2.7. Work Engagement

Work engagement has been discussed as both a potential antecedent and a potential outcome of technology use. Some studies suggest that engaged employees are more willing to experiment with digital tools, whereas other work indicates that AI use itself can raise or lower engagement depending on how AI affects core tasks and meaning at work (Faida et al., 2023; Liu & Li, 2025; Rick et al., 2024).

Accordingly, we treat engagement as an exploratory correlate rather than as a clearly causal predictor. Although UTAUT-based extensions have included engagement-like constructs, work engagement is not part of the original UTAUT model (Venkatesh et al., 2003). We therefore examine whether engagement is associated with AI use in the current sample, while recognizing the likely bidirectionality of this relationship.

## 3. Method

### 3.1. Participants and Procedure

The sample consisted of 196 employees in Norway, aged 18 years or older. ata were collected with an anonymous online survey using Nettskjema, a secure survey platform provided by the University of Oslo (Oslo, Norway), between 22 November 2024 and 31 January 2025. Complete anonymity was ensured by disabling the collection of IP addresses or other identifiers, and no survey items requested personal data. Informed consent was obtained before participation. The survey was distributed via social media, email to organizations, and personal networks. No personal data was collected, and thus approval from the Norwegian Agency for Shared Services in Education and Research (Sikt) was not required. Because recruitment relied on voluntary response and personal networks, the sample should be regarded as a convenience sample. This is especially important for the present topic, as respondents with greater interest in or exposure to AI may have been more likely to participate, potentially biasing upward the observed rate of AI use. Gender was measured by self-report. In the analytic sample, responses were coded as male or female, and one respondent did not report gender and was treated as missing in analyses involving gender.

### 3.2. Instruments

Validated scales were used to assess SBL and work engagement. Perceived work training (M = 4.47, SD = 1.11) was measured with the single item: “In my organization, I received sufficient training to perform my work tasks in a proper manner.” This item captures perceived adequacy of general job training rather than AI-specific training or AI literacy and is therefore interpreted cautiously. All scales were forward- and back-translated (Brislin, 1970). SBL (M = 5.09, SD = 1.20) was measured with the 8-item scale by J. Wang et al. (2023), adapted from Keenan and Mostert (2013). The scale is answered on a 7-point Likert scale (1 = strongly disagree, 7 = strongly agree). The original scale showed very high internal consistency (α = 0.97; J. Wang et al., 2023), and the translated scale showed α = 0.94 in the current study.

Work engagement (M = 5.28, SD = 1.41) was measured with the UWES-3 (Schaufeli, 2003; Schaufeli et al., 2019). The three items capture vigour, dedication, and absorption, rated from 0 (never in the past year) to 6 (daily). In other studies, internal consistency ranges from acceptable to excellent: α = 0.77 in a nationally representative Korean worker sample (An et al., 2020), α = 0.94 in a Peruvian worker sample (Merino-Soto et al., 2022), and α = 0.95 in a Dutch workforce sample (Schaufeli et al., 2019). Reliability of the Norwegian version in the current study was good (α = 0.85).

### 3.3. Data Analysis

All analyses were conducted using IBM SPSS Statistics, version 30 (IBM Corp., Armonk, NY, USA). Statistical significance was evaluated at *p* < 0.05 (two-tailed).

We first report the overall percentage of AI users and examine AI use across sociodemographic and occupational variables (education, age group, sector, gender, leadership role). For categorical comparisons, Pearson’s χ^2^ tests were applied, with Fisher’s exact test used in 2 × 2 tables and Monte Carlo correction in tables with sparse cells. For continuous variables (work training, SBL, and engagement), independent samples t-tests were performed to compare AI users and non-users. Levene’s test determined whether equal variances could be assumed. Effect sizes were reported as Cohen’s d with 95% confidence intervals.

To examine associations with self-reported AI use, we estimated hierarchical logistic regression models. AI use (0 = non-user, 1 = user) was the dependent variable. Model 1 included the sociodemographic variables of education, age group, gender, leadership role, and work sector. Model 2 added perceived work training, SBL, and work engagement to assess whether organizational and psychological variables contributed beyond sociodemographic and sectoral factors, irrespective of their bivariate significance.

Given the modest sample size and sparse cells in some education and sector categories, we also estimated an exploratory reduced model with collapsed education and sector categories. Education was recoded into ≤ upper secondary, bachelor’s degree, and master’s degree or higher; sector was recoded into knowledge-intensive versus skills-focused work. Industry (*n* = 2) and the heterogeneous “Other” category were excluded from this reduced model. Because this model differs in coding from Models 1 and 2, we interpret it as exploratory rather than as the primary inferential model. As an additional robustness check, we estimated a simultaneous model using the recoded education and sector variables together with age, gender, leadership role, training, SBL, and engagement. We also inspected multicollinearity using variance inflation factors (VIF). VIF values ranged from 1.03 to 2.62, indicating no problematic multicollinearity. Across all analyses, results are interpreted as associations rather than causal effects.

## 4. Results

### 4.1. Descriptive Statistics

The analytic sample consisted of 196 respondents, of whom 1 did not report gender (Table 1). Overall, 143 participants reported using AI at work, and 53 did not. Most respondents were women (115 vs. 80 men), and 19 held a leadership role. Age groups were distributed as 77 aged 18–34 years, 59 aged 35–49, and 60 aged 50 years and older. Regarding education, two had completed only primary school, 21 had completed upper secondary school, 51 had a bachelor’s degree, and 122 had a master’s degree. Participants came from several sectors, although the distribution was concentrated in public administration (*n* = 40), IT and media (*n* = 36), health and care (n = 30), and education (*n* = 24) rather than evenly spread across sectors. The high observed AI-use rate (72.9%) should therefore be interpreted in light of the convenience-sample design.

### 4.2. Chi-Square Tests

Chi-square tests were performed to explore demographic differences in AI use (Table 2). AI use was significantly associated with gender, χ^2^(1, N = 195) = 4.35, *p* = 0.037, φ = 0.15, with men reporting higher use (81%) than women (68%). No significant differences were observed across age groups, χ^2^(2, N = 196) = 1.81, *p* = 0.405, V = 0.10. Education was strongly associated with AI use, χ^2^(3, N = 196) = 29.42, *p* < 0.001, V = 0.39, with bachelor’s and master’s degree holders most likely to report use. Leaders reported higher use (90%) than non-leaders (71%), but this difference was not statistically significant, χ^2^(1, N = 196) = 2.91, *p* = 0.088, φ = 0.12. AI use also varied by sector, χ^2^(9, N = 196) = 39.26, *p* < 0.001, V = 0.45, with IT, finance, and education showing the highest rates. For education and sector, Monte Carlo simulations with 10,000 samples and a 95% confidence interval confirmed the robustness of these bivariate results.

### 4.3. Independent Sample t-Tests

Independent samples t-tests compared AI users and non-users on work training, SBL, and work engagement (see Table 3). No differences were observed for work training, t(194) = 0.02, *p* = 0.986, d = 0.00, or work engagement, t(194) = 0.04, *p* = 0.968, d = 0.01. However, AI users reported significantly higher SBL (M = 5.25, SD = 0.91) than non-users (M = 4.65, SD = 1.32), Welch’s t(77.34) = –2.87, *p* = 0.005, d = 0.52, indicating a medium effect. Equal variances were assumed for work training and engagement. Welch’s correction was used for SBL due to unequal variances.

### 4.4. Logistic Regression Analyses

To examine predictors of AI use, we conducted three logistic regression models. Model 1 included sociodemographic variables (age, gender, leadership position, education, and sector). The model was significant, χ^2^(16) = 71.75, *p* < 0.001, with Nagelkerke R^2^ = 0.448. It correctly classified 81.5% of cases, although this should be interpreted relative to the 72.9% base rate of AI users in the sample. Hosmer–Lemeshow indicated acceptable fit, χ^2^(8) = 7.25, *p* = 0.51. Education, sector, and gender emerged as significant predictors (see Table 4). However, interpretation of the sector and education coefficients is limited by sparse cells and quasi-separation, especially for the primary-school and finance categories.

Model 2 added the organizational context variables as a block (Table 5). Adding work training, SBL, and engagement improved model fit, χ^2^(3) = 10.67, *p* = 0.014. The overall model was significant, χ^2^(19) = 82.42, *p* < 0.001, Nagelkerke R^2^ = 0.50, with 81.0% classification accuracy and acceptable Hosmer–Lemeshow fit, χ^2^(8) = 4.71, *p* = 0.79. SBL was positively associated with AI use (OR = 2.03). Gender and education remained significant, whereas sector effects were attenuated once organizational variables were included, suggesting overlap between sectoral context and the other predictors.

To address sparse cells, an exploratory reduced model was estimated using collapsed education and sector variables (Table 6). This model is not directly comparable to Models 1 and 2 because education and sector were recoded and small heterogeneous categories were excluded. It is therefore presented as a descriptive summary of the main pattern rather than as the primary inferential basis of the article.

In this reduced model, SBL, gender, education, and sector type were associated with AI use. Each one-point increase in SBL was associated with higher odds of AI use (OR = 1.89). Employees in knowledge-intensive sectors were about 2.5 times more likely to use AI than those in skills-focused sectors (OR = 2.52). Women had substantially lower odds than men (OR = 0.34). Education showed the strongest gradient: compared to respondents with upper secondary education or less, those with a bachelor’s degree were more likely to report AI use (OR = 3.64), and those with a master’s degree or higher were more likely still (OR = 11.15). Given the skewed sample and small cells in some original categories, these effect sizes should be interpreted cautiously.

## 5. Discussion

This study examined associations between self-reported workplace AI use and individual, sectoral, and organizational factors in a Norwegian employee sample. Across models, higher education, work in knowledge-intensive sectors, male gender, and higher perceived SBL were the most consistent correlates of AI use. However, it should be noted that the study does not measure adoption as a broader process of acceptance and implementation; rather, it captures whether employees reported using AI at work. The findings should therefore be read as exploratory associations with self-reported AI use, not as causal evidence that these factors drive workplace AI use.

The absence of an effect for leadership role is also informative. Simply holding a leadership position was not associated with AI use, whereas perceived leadership style was. This distinction suggests that positional status alone may matter less than employees’ day-to-day experience of supportive leadership behaviors. Still, given the small number of leaders in the sample (n = 19), this result should be treated as tentative. The strongest pattern concerned education. Employees with higher formal education were more likely to report AI use, and this gradient persisted after accounting for sector and leadership variables. This aligns with prior research linking education to digital literacy, problem-solving capacity, and access to knowledge-intensive tasks (Bick et al., 2024; Kacperski et al., 2025; Lane, 2024; Lane et al., 2023). At the same time, the large odds ratios observed here should be interpreted cautiously. In a sample that is both highly educated and relatively small, education may also capture unmeasured occupational and task-level differences in exposure to AI, not only a direct educational advantage. The robustness model suggests that the clearest education contrast is concentrated at the master’s-or-higher level, whereas the bachelor’s-degree contrast was less stable once the recoded predictors were estimated simultaneously.

A second pattern concerned sector. Employees in knowledge-intensive sectors were more likely to report AI use, but the meaning of this effect is constrained by the study’s sector operationalization. Sector is a coarse proxy that likely captures differences in task feasibility, AI exposure, and occupational composition rather than an industry effect in any strong causal sense. The attenuation of sector effects from the bivariate analyses to the multivariable models suggests overlap with education and other predictors. Accordingly, the sector finding is better interpreted as evidence that AI use is more common in task environments where current tools are more applicable (Bick et al., 2024; Eurostat, 2024; Handa et al., 2025; Tomlinson et al., 2025).

The most novel aspect of the study concerns SBL. Perceived SBL remained positively associated with AI use even after other predictors were included. This does not demonstrate that SBL causes AI use, nor does it establish self-efficacy as the operative mechanism. Rather, the finding suggests that a strengths-oriented leadership climate may matter alongside demographic and sectoral differences in a highly digitalized Norwegian context. This is a modest contribution, but it is potentially useful because the literature on workplace AI has focused more heavily on structural factors than on specific leadership styles.

The gender difference also remained visible across models. Men reported higher AI use than women, and this difference persisted after adjustment for education and sector. However, the present study does not measure the mechanisms that might account for this pattern. Unmeasured differences in AI exposure, task composition, norms, confidence, or AI-related anxiety may all be relevant. The finding should therefore not be read as evidence for any one cultural or psychological explanation, but as a descriptive inequality that warrants more targeted investigation (Aldasoro et al., 2024; Conde-Ruiz et al., 2024; Otis et al., 2024; Russo et al., 2025).

Age was not associated with AI use once the other variables were considered. This differs from much of the existing literature, which often reports lower use among older employees (Da Silva & Weißler, 2025; Dingel et al., 2024; Fazi et al., 2025; Y. Kim et al., 2024; SSB, 2025). One possibility is that the broad age categories used here did not capture sufficient variation. Another is that age effects in workplace AI use are partly absorbed by education, sector, and organizational context. The present data do not allow these explanations to be disentangled.

### 5.1. Limitations

Several limitations should be acknowledged. First, the study used a cross-sectional convenience sample from Norway, a highly digitalized context, which limits external validity and may upwardly bias reported AI use if AI-interested employees were more likely to respond. Second, all variables were self-reported at one time point, raising the possibility of common method bias despite the anonymous survey design. Third, the dependent variable captured binary self-reported AI use rather than frequency, depth, voluntariness, or specific task applications. As such, the measure may partly reflect whether respondents had the opportunity or task conditions to use AI at work, not only whether they were personally inclined to use it. Fourth, the single training item captured general job training rather than AI-specific training or AI literacy. Finally, sparse cells, quasi-separation, and the need to collapse categories constrain precision.

Future research would benefit from longitudinal or quasi-experimental designs, probability samples, multi-source leadership ratings, and richer measures of AI use, training, task demands, and organizational context. Cross-national comparisons would also be valuable for assessing whether the Norwegian pattern generalizes to less digitalized settings or to workplaces with different institutional arrangements.

### 5.2. Implications

These findings suggest cautious practical implications for organizations seeking to support workplace AI use (Arranz Lahuerta et al., 2026). First, the education pattern indicates that AI use may follow existing skill divides, which may justify tailored enablement strategies for employees with different backgrounds. Second, the gender gap points to the importance of inclusive access to tools, guidance, and opportunities, but the present study does not identify the mechanisms that would justify narrowly targeted interventions. Third, the positive association with SBL suggests that leadership development focused on supportive, strengths-oriented behaviors may be worth evaluating as part of broader digital transformation efforts. Finally, the null result for general work training should not be interpreted as evidence against AI-specific training; rather, it suggests that broad, non-specific training perceptions are unlikely to be sufficient on their own. More broadly, these findings suggest that how AI is introduced and supported at work may have implications beyond immediate tool use, although the present study was not designed to test downstream behavioral outcomes.

## 6. Conclusions

In conclusion, self-reported workplace AI use in this Norwegian convenience sample was most consistently associated with higher education, employment in knowledge-intensive sectors, male gender, and stronger perceived SBL. Robustness analyses suggested that this overall pattern was largely stable, while also indicating that the clearest education contrast was concentrated at the master’s-or-higher level. Age, leadership role, general work training, and work engagement were not reliably associated with AI use in this sample. Because the study relied on cross-sectional self-reports, a binary measure of AI use, and models affected by sparse cells, these results should be read as exploratory associations rather than evidence that these factors explain reported workplace AI use in a causal sense. The main contribution is therefore modest: organizational context, and particularly perceived SBL, may help explain variation in reported AI use beyond demographic and sectoral differences, but this interpretation requires replication with larger and more precise designs.

## Figures and Tables

**Table 1:** Distribution of Demographic Variables.

Variable	Category	n
AI use	No	53
	Yes	143
Gender	Male	80
	Female	115
	Missing	1
Leadership	No	177
	Yes	19
Age	18–34	77
	35–49	59
	50+	60
Education	Primary school	2
	Upper secondary school	21
	Bachelor’s degree	51
	Master’s degree	122
Sector	Health and care	30
	Public administration	40
	IT and media	36
	Finance and insurance	18
	Education	24
	Other	24
	Professional services	7
	Trade and services	4
	Construction	11
	Industry	2

Note. One case is missing for gender.

**Table 2:** Chi-Square Tests of AI Use by Demographic Variables.

Predictor	χ^2^(df)	*p*	Effect Size
Gender	4.35(1)	0.037 a	φ = 0.15
Age	1.81(2)	0.405	V = 0.10
Education	29.42(3)	<0.001 b	V = 0.39
Leadership role	2.91(1)	0.088 c	φ = 0.12
Sector	39.26(9)	<0.001 b	V = 0.45

Notes. Pearson χ^2^ reported; effect size is φ for 2 × 2, Cramér’s V otherwise. a, Fisher’s exact (two-sided) also inspected for robustness: *p* = 0.048. b, Due to sparse cells, Monte Carlo exact *p*-values were computed with 10,000 samples and 95% confidence level; conclusions unchanged (*p* < 0.001). c, For leader (2 × 2) with borderline expected counts, Fisher’s exact (two-sided) = 0.107.

**Table 3:** Independent Samples t-Tests Comparing AI Users and Non-Users on Work Training, SBL, and Work Engagement.

Variable	Group	n	M	SD	t(df)	*p*	Cohen’s d
Work Training	Non-users	53	4.47	1.12	0.02(194)	0.986	0.00
	AI users	143	4.47	1.11			
SBL	Non-users	53	4.65	1.32	–2.87(77.34)	0.005	0.52
	AI users	143	5.25	0.91			
Work engagement	Non-users	53	5.29	1.54	0.04(194)	0.968	0.01
	AI users	143	5.28	1.36			

Note. Means (M) and standard deviations (SD) are reported per group. Equal variances were assumed for training and work engagement, while Welch’s correction was applied for SBL due to unequal variances. Cohen’s d uses pooled standard deviation.

**Table 4:** Logistic Regression Predicting AI Use—Model 1 (Sociodemographics).

Predictor	B	SE	OR	95% CI	*p*
Leader	1.36	0.98	3.89	[0.56, 26.44]	0.172
Age 35–49	0.71	0.55	2.04	[0.69, 5.99]	0.196
Age 50+	−0.27	0.50	0.77	[0.29, 2.04]	0.592
Gender (Female)	−1.03	0.45	0.36	[0.15, 0.86]	0.021
Education (overall)	—	—	—	—	<0.001
Public Admin	0.22	0.62	1.25	[0.37, 4.18]	0.718
IT & Media	2.60	0.78	12.93	[2.87, 59.15]	<0.001
Finance	—	—	—	—	0.998
Education sector	0.84	0.74	2.32	[0.55, 9.80]	0.253
Other	1.46	0.75	4.31	[1.00, 18.58]	0.050
Prof. Services	0.16	1.05	1.18	[0.15, 9.23]	0.878
Retail/Service	1.85	1.50	6.36	[0.33, 121.14]	0.219
Construction	0.94	0.97	2.56	[0.38, 17.14]	0.333
Industry	−0.90	2.00	0.41	[0.01, 20.48]	0.653

Note. Categorical coding: Leader (1 = leader, 0 = non-leader); Gender (1 = female, 0 = male). Finance dummy estimates showed quasi-separation (perfect prediction) due to very small cells. Leader reference = non-leader; Age reference = 18–34; Gender reference = male.

**Table 5:** Logistic Regression Predicting AI Use—Model 2 (Added Occupational Variables).

Predictor	B	SE	OR	95% CI	*p*
Leader	1.08	1.06	2.95	[0.37, 23.47]	0.307
Age 35–49	0.50	0.58	1.65	[0.53, 5.13]	0.391
Age 50+	−0.54	0.54	0.58	[0.20, 1.68]	0.317
Gender (Female)	−1.08	0.45	0.34	[0.14, 0.83]	0.017
Education (overall)	—	—	—	—	<0.001
Public administration	−0.08	0.66	0.92	[0.25, 3.38]	0.899
IT & media	2.35	0.81	10.53	[2.14, 51.82]	0.004
Finance & insurance	—	—	—	—	0.998
Education sector	0.94	0.81	2.55	[0.52, 12.43]	0.248
Other	1.26	0.76	3.54	[0.79, 15.79]	0.098
Professional services	−0.25	1.08	0.78	[0.09, 6.48]	0.815
Retail/Service	1.71	1.40	5.55	[0.36, 85.51]	0.219
Construction	0.81	1.02	2.24	[0.31, 16.41]	0.426
Industry	−0.75	2.14	0.47	[0.01, 31.47]	0.727
Work training	−0.34	0.23	0.72	[0.46, 1.12]	0.146
SBL	0.71	0.24	2.03	[1.28, 3.23]	0.003
Engagement	−0.05	0.17	0.95	[0.68, 1.33]	0.774

Note. Categorical coding: Leader (1 = leader, 0 = non-leader); Gender (1 = female, 0 = male); Age reference = 18–34; Sector reference = Health & care. Finance & insurance shows quasi-separation. OR = odds ratio; CI = confidence interval.

**Table 6:** Logistic Regression Predicting AI Use—Model 3 (Recoded Education, Knowledge Sector).

Predictor	B	SE	OR	95% CI	*p*
Strengths-based leadership	0.64	0.17	1.89	[1.35, 2.64]	<0.001
Knowledge sector	0.92	0.42	2.52	[1.12, 5.68]	0.026
Gender (Female)	−1.09	0.44	0.34	[0.14, 0.80]	0.013
Education (overall)					<0.001
3 years	1.29	0.60	3.64	[1.12, 11.91]	0.032
5 years +	2.41	0.58	11.15	[3.59, 34.92]	<0.001

Note. Reference categories were ≤ upper secondary education, skills-focused sectors (health & care, professional services, retail/trade & services; industry and “other” were excluded), and male (for gender). Education and sector were entered as categorical variables with dummy coding. Because Model 3 uses recoded variables and excludes small categories, it should be interpreted as exploratory. OR = odds ratio; CI = confidence interval.

## Data Availability

The data presented in this study are available on request from the corresponding author.
